# Biodiversity risks and corporate innovation: Evidence from China

**DOI:** 10.1371/journal.pone.0342348

**Published:** 2026-03-18

**Authors:** Yongjian Lin, Zhicheng Song

**Affiliations:** School of Economics and Management, Xiamen university of technology, Xiamen, China; USTC: University of Science and Technology of China, CHINA

## Abstract

Whether firms adopt positive behaviors in response to biodiversity risk is a matter of public concern. In this paper, we investigate the impact of biodiversity risk exposure on corporate innovation. Based on a sample of Chinese A-share listed firms from 2007–2022, we find that biodiversity risk is significantly and positively related to corporate innovation. This promotion can be attributed to higher operating costs and enhanced analyst attention. Meanwhile, the positive impact of biodiversity risk on corporate innovation is more pronounced in firms with high investor sentiment, non-high-tech industries, and more liberalized capital markets. Additionally, the promotion of innovation by biodiversity risk is manifested more in substantive innovation. This study enriches the research on the consequences of biodiversity risk and the factors influencing firm innovation, expanding the boundaries of how environmental risk affects corporate decision-making.

## Introduction

Biodiversity refers to the sum of genes, species, and ecosystems [1]. In turn, biodiversity risk is the physical and transformation risk to the value of associated economic activities and assets caused by biodiversity loss [2]. Biodiversity risk fundamentally differs from climate risk [2]. However, in contrast to climate risk, an actively researched field [3,4], studies related to biodiversity risk are still relatively scarce at present. Indeed, the biodiversity degradation has equally significant impacts on the economic activities of human societies [5]. It is estimated that biodiversity degradation in recent years has caused annual losses ranging from about $4 trillion to $20 trillion [6]. In terms of micro impacts, some researchers have found that biodiversity risk reduces corporate operational efficiency and increases the precautionary cash-holding incentives of firms [6,7]. Moreover, biodiversity risk has affected corporate capital market performance [8,9]. Consequently, in recent years, governments and international institutions around the globe have become increasingly aware of the challenges that biodiversity poses to sustainable economic development.

In 2021, the fifteenth Conference of the Parties to the United Nations Convention on Biological Diversity adopted the Kunming Declaration, calling on all countries to take action to respond to the call for curbing the loss of biodiversity [10]. In the same year, the Chinese government issued the white paper Biodiversity Conservation in China [11]. The document states that, facing global biodiversity loss and ecosystem degradation, China commits to establishing a government-led biodiversity conservation mechanism characterized by broad public participation and multi-stakeholder governance. Against the background that the government attaches great importance to biodiversity conservation, this paper attempts to explore the response of enterprises from the perspective of technological innovation. The reason is that technological innovation is a powerful weapon for preventing major risks and supporting sustainable development. Meanwhile, the Kunming-Montreal Global Biodiversity Framework emphasizes that capacity building, technical cooperation, and knowledge sharing are crucial for biodiversity conservation in developing countries [12]. For example, agriculture, aquaculture, fisheries, and forestry can protect biodiversity through innovative approaches such as sustainable intensification and agroecology.

This paper argues that understanding the relationship between biodiversity risk and firm innovation may have both positive and negative implications. On the one hand, external pressures may incentivize firms to innovate [13]. Advances in corporate technology can lead to higher natural resource utilization and lower rates of environmental pollution [14–16]. This reduces the probability of firms being exposed to resource shortages as well as regulatory penalties. Concurrently, firms also increase social benefits and generate an ethical insurance effect [17,18]. Therefore, firms have the prospect of increasing technological innovation to counter the challenges posed by the risk of biodiversity loss. On the other hand, innovation activities feature greater uncertainty and risk, and long payoff cycles [19]. Faced with the risk of biodiversity loss, firms may cut R&D investment for the purpose of controlling total risk [20]. Consequently, the question of whether biodiversity risk promotes or inhibits firms’ innovation activities remains to be empirically tested.

This paper seeks to illustrate the theoretical mechanisms through which biodiversity risk influences corporate innovation, and conducts an empirical study targeting listed companies in China. Unlike the existing research finding that biodiversity risk exposure causes firms to operate conservatively [6], this paper finds that there is a significant positive relationship between corporate biodiversity risk exposure and the number of patent applications. The mechanism test finds that biodiversity risk raises the operating costs of firms and also attracts the attention of analysts, thereby promoting corporate innovation. Meanwhile, the degree of influence of corporate biodiversity risk exposure is related to investor sentiment, industry characteristics, and capital market environments. Additionally, the promotion of innovation by biodiversity risk is manifested more in substantive innovation.

This paper’s main contributions include three aspects. First, this paper enriches the literature on corporate innovation decisions from a biodiversity risk perspective. The impact of social environmental risks and natural environment risks on firms’ innovation has been extensively explored in the established literature. Social environmental risks include geopolitical risks [21], cybersecurity risks [22], and litigation risks [23], among others. Regarding natural environmental risks, scholars have focused on the impact of climate change or climate disasters on firms’ innovation [24–26]. Evidently, little literature has explored the relationship between biodiversity risk and corporate innovation. Biodiversity loss can cause great harm and is a chronic and abstract risk [27]. Accordingly, this paper emphasizes that natural environmental risks should be fully considered in addition to the climate risk topic, and that the impact of biodiversity risk on business decisions should be taken into account.

Second, by examining how biodiversity risk influences firm innovation, we add to the emerging literature on economic issues related to natural resources. Prior work on biodiversity has emphasized its social value [27] and paid less attention to the economic implications [2]. Scholars have only recently begun to focus on the economic impact of biodiversity risks on firms [28]. Ahmad and Karpuz [6], Li et al. [7], and Bach et al. [29] examined the impacts of biodiversity risk on corporate operational efficiency and cash decisions. Bassen et al. [30] and Liang et al. [8] explore how biodiversity risk affects firms’ stock value. We support the theory of strategic growth options by examining how biodiversity risk affects firms’ innovations, providing new perspectives for recognizing firms’ strategic responses to biodiversity risk. Meanwhile, the analysis in this paper has critical practical implications since corporate innovation can further affect macroeconomic growth [31] and corporate long-term competitive advantage [32].

Third, it contributes to comparing and deepening relevant research on the relationship between biodiversity risk and corporate decision-making in different market scenarios. Existing studies are mainly limited to developed countries represented by the United States [6,7,29], and there is a lack of attention to emerging markets. China, as the world’s largest developing country and the world’s second largest economy, has the dual characteristics of emerging plus transition. The market environment and governance situations faced by Chinese firms, such as industrial policies, capital market maturity, and agency problems, are considerably different from those of countries with mature markets [33]. Consequently, this study, based on the Chinese scenario, can provide unique and important empirical evidence for understanding the impact of biodiversity loss on emerging markets.

## Hypothesis development

This paper argues that biodiversity risk may promote firm innovation by increasing the operating costs of firms and attracting analyst attention for the following reasons:

First, biodiversity risk can significantly increase firms’ resource acquisition costs. At first, biodiversity, as an integral part of the natural capital stock, is exposed to risks that can cause a reduction in natural capital inputs [29]. According to neoclassical economic growth theory, reductions in factor inputs will result in a decline in total firm output [34,35]. Thus, enterprises exposed to biodiversity risk require increased unnatural capital inputs to maintain output levels [36], ultimately increasing operating costs. Second, biodiversity risk exposure will lead to firms being confronted with transition risks caused by regulatory changes, including regulatory penalties and reputational damage [37]. This may entail firms being boycotted by consumers and investors, and ultimately translate into higher costs of sales and financing for firms. Furthermore, biodiversity risks may also disrupt the firm’s supply chain [2], thereby increasing the firm’s operating costs.

Higher operating costs and the long-term nature of biodiversity risk will motivate firms to seek innovation. Based on the strategic growth option theory, external shocks will weaken the value of the waiting option and encourage firms to increase strategic investments with growth option value [38]. The long-term nature of biodiversity risk will persist as governments continue to focus on environmental issues [39]. This means that the increase in business operating costs due to biodiversity risks will be persistent. To cope with the increase in operating costs, more firms will choose to innovate by developing new products and business models to gain the first mover advantage while compensating for environmental management costs [40].

Second, biodiversity risk can attract analysts’ attention. As the popularity of ESG concepts accelerates, biodiversity risk is emerging as a cutting-edge topic in financial risk management. Existing literature demonstrates that the uncertainty and legal risks arising from the biodiversity issue have entered the risk assessment framework of investors [41] and that this has significantly impacted stock returns [9]. Accordingly, analysts may pay closer attention to firms exposed to biodiversity risk for a deeper investigation of the stock market.

As crucial market intermediaries, analysts are essential mechanisms for the external governance of capital markets [42,43]. Particularly, investors in emerging markets are dominated by retail investors with information disadvantages [44], and there is a stronger requirement for specialized market intermediaries to exploit effective information. Furthermore, analyst concerns may improve the information environment inhabited by firms, thereby affecting firms’ innovation decisions under information asymmetry. First, innovation activities feature high professionalism, confidentiality, and uncertainty, making it extremely difficult for investors to precisely assess the enterprise’s prospective returns, and consequently undervaluing firms with more innovative activities [45]. Analysts interpret corporate information based on their specialized knowledge and skills, enhancing corporate information transparency to some extent [46]. Ultimately, it reduces the resource mismatch in the capital market [47] and improves firms’ willingness to invest in innovation. Second, the management of high agency cost firms tends to consider the personal benefits from short-term performance, and their business behavior is more short-sighted, thus their preference for R&D investment tends to be lower [48]. Whereas analysts, through the information transfer mechanism, can play a certain inhibiting effect on the short-sighted behavior of agents [49]. Thereby, it will prompt the management to take a longer-term view of decision-making and eventually increase the firm’s innovation investment. Based on the above analysis, this paper proposes the following hypotheses:

**Hypothesis 1.** Biodiversity risk can motivate corporate innovation.

**Hypothesis 1a.** Biodiversity risk can motivate corporate innovation by increasing the corporate operating costs.

**Hypothesis 1b.** Biodiversity risk can motivate corporate innovation by attracting analysts’ attention.

Real options theory, resource dependence theory, and rent-seeking theory provide new horizons for understanding the connection between biodiversity risk and corporate innovation. First, owing to the irreversibility of investment, external risks may enhance the waiting value of options [50]. Hence, confronted with the physical and transition costs of biodiversity risk, companies may choose to increase their cash reserves to retain flexibility in their future investment decisions [6]. This will have a crowding-out effect on firms’ R&D investment. Second, investors and creditors may lose confidence in biodiversity-risky firms because of the greening requirements of the economy, negatively affecting the stock value and financial position of the firms [41], and subsequently exacerbating the financing constraints. According to resource dependence theory, firm development is largely shaped by the capacity to access external resources [51]. When financing constraints are aggravated, firms may be inclined to cut back on non-essential expenditures, including R&D investment [52]. Third, in developing countries where financial development is backward and government power is concentrated, enterprises are more likely to establish political relationships with a view to obtaining more government resources. Accordingly, firms in emerging markets may engage in rent-seeking activities to minimize the intensity of environmental regulations, thereby crowding out R&D resources [53]. In summary, biodiversity risks may reduce the willingness and ability of corporate innovation. Based on the above analysis, this paper proposes the following hypotheses:

**Hypothesis 2.** Biodiversity risk can inhibit corporate innovation.

## Design development

### Sample selection and data source

In 2006, China’s accounting standards for enterprises underwent major changes, which made the disclosure of relevant financial data by listed companies more systematic. Therefore, we choose Chinese listed companies from 2007 to 2022 as the initial sample and follow the following principles to screen the sample: (1) excluding listed companies with missing in the main variables; (2) excluding listed companies in the financial category; (3) excluding ST and PT companies; and (4) Winsorize shrinkage of continuous variables with greater than 99% and less than 1% quantile. The final research sample obtained after processing contains 31,151 observations. Biodiversity risk data at the enterprise and industry levels are sourced from He et al. [37] and Chen et al. [54], respectively. Other data mainly comes from the China Stock Market & Accounting Research Database and the Chinese Research Data Services Platform.

### Model building

We examine the impact of biodiversity risk on firm innovation and construct the ordinary least squares (OLS) model:


$$PAT_{i,t}+\beta_{\text{0}}+\beta_{\text{1}}BR_{i,t}+\beta_{\text{2}}Controls_{i,t}+YEAR+FIRM+\varepsilon_{i,t}$$ (1)

Where i denotes the firm, t denotes the year. PAT denotes the innovation output of the firm, BR is a dummy variable for whether the firm is exposed to biodiversity risk, Control is the control variable selected in this paper, YEAR and FIRM denote the year fixed effect and the individual firm fixed effect, and εi,t is the error term. Standard errors are clustered at the firm level.

### Primary Variables

#### Dependent variable: Corporate innovation.

We refer to the existing literature [55–58] on the use of patent-based metrics to access firm innovation. According to Pakes et al. [59], patents are granted with a time lag, so we measure the number of patents based on the year of filing. Since the distribution of the number of innovations is highly skewed, we take the natural logarithm of all patent numbers after adding 1 in this paper to finally obtain the variable PAT.

#### Independent variable: Biodiversity risks.

To measure biodiversity risk at the enterprise level, we adopted the text-based enterprise-specific biodiversity risk measure of Giglio et al. [2] and He et al. [37]. First, the authors build a biodiversity lexicon to identify texts that cover biodiversity-related topics. See the appendix for specific details. Second, the authors conducted text mining to count the frequency of these biodiversity terms based on the annual reports published by the companies. Finally, the Biodiversity Risk Index (BR) was assigned a value of 1 if the term frequency was more than two occurrences and 0 otherwise.

#### Control variables.

We refer to the existing literature [54,60,61] and select the following control variables, including SIZE, LEV, ROA, GROWTH, BOARD, TOBINQ, FIXED, GS, and GDP. Table 1 provides the detailed definitions of the main variables.

**Table 1:** Definitions of main variables.

Variable	Definition
PAT	The number of patents plus one is taken as the natural logarithm
BR	Corporate biodiversity risk exposure
SIZE	The total value of enterprise assets is taken as a natural logarithm
LEV	Ratio of total liabilities to total assets
ROA	The ratio of net income over total assets
GROWTH	The ratio of total asset growth at the end of the year over the total assets at the beginning of the year
BOARD	The natural logarithm of board size
TOBINQ	Ratio of market value to book value of the enterprise
FIXED	Ratio of fixed assets to total assets
GS	Ratio of government grants to total assets
GDP	The natural logarithm of gross domestic product

## Empirical results and analysis

### Descriptive statistics

Table 2 shows the descriptive statistics. The mean value of BR is 0.456, indicating that Chinese A-share companies are generally exposed to biodiversity risk. The standard deviation of PAT is 1.74, indicating that there is a large disparity in innovation outputs among firms.

**Table 2:** Descriptive statistics.

Variable	Obs	MEAN	P50	SD	Min	Max
PAT	31151	2.61	2.773	1.74	0	6.756
BR	31151	0.456	0	0.498	0	1
SIZE	31151	22.227	22.035	1.287	19.867	26.186
LEV	31151	3.352	2.364	2.88	1.127	19.611
ROA	31151	0.042	0.039	0.063	−0.208	0.228
GROWTH	31151	0.167	0.111	0.377	−0.541	2.343
BOARD	31151	2.131	2.197	0.2	1.609	2.708
TOBINQ	31151	2.04	1.634	1.27	0.854	8.119
FIXED	31151	0.214	0.182	0.159	0.002	0.699
GS	31151	0.005	0.003	0.005	0	0.028
GDP	31151	18.144	18.259	1.137	15.262	19.917

### Baseline results

To test whether biodiversity risk affects corporate innovation, we run regressions based on equation (1). As shown in Table 3, column (1) reports the results controlling for firm and year fixed effects but not firm-level characteristic variables, while column (2) shows the results after adding control variables. The results show that in both regressions, the estimated coefficient on BR is significantly positive, supporting Hypothesis 1. Analyzing the economic significance, the coefficient on PAT is 0.0573, which infers that biodiversity risk can, on average, increase firms’ innovation by 2.20% (0.0573/2.61).

**Table 3:** Biodiversity risks and corporate innovation.

	(1)	(2)
Variable	PAT	PAT
BR	0.1251***	0.0573***
	(0.0206)	(0.0194)
SIZE		0.4852***
		(0.0285)
LEV		0.0022
		(0.0047)
ROA		0.1318
		(0.1609)
GROWTH		−0.0165
		(0.0171)
BOARD		0.1445*
		(0.0768)
TOBINQ		0.0114
		(0.0088)
FIXED		0.0983
		(0.1334)
GS		11.0548***
		(1.9369)
GDP		0.0975
		(0.0602)
Constant	2.5533***	−10.3826***
	(0.0094)	(1.2261)
Year/Firm FE	Yes	Yes
Observations	31151	31151
Adj-R2	0.7360	0.7501

Note: *, **, and *** denote significant at 10%, 5%, and 1% levels, respectively.

### Robustness checks

#### Instrumental variable.

We cite Feng et al. [62] to use the average biodiversity risk (IV) of other firms in the same industry as an instrumental variable. Table 4 shows the results of the two-stage least squares (IV-2SLS) estimation. Column (1) shows that the IV coefficient is significantly positive, suggesting that IV is correlated with firms’ biodiversity risk exposure. Column (2) of Table 4 shows that biodiversity risk exposure is positively associated with firm innovation.

**Table 4:** Robustness checks.

	(1)	(2)	(3)	(4)	(5)	(6)
VARIABLES	BR	PAT	PAT	PAT	PAT	PAT
BR		0.5662***		0.0572***	0.0547***	0.0077 (0.0176)
		(0.1926)		(0.0194)	(0.0199)	0.0077 (0.0176)
IV	0.3176***					
	(0.2304)					
IMR				1.3234**		
				(0.5988)		
SIZE	0.0877***	0.4383***	0.3165***	0.7508***	0.4878***	0.4205***
	(0.0053)	(0.0239)	(0.0139)	(0.1272)	(0.0297)	(0.0092)
LEV	−0.0017	0.0032	0.0002	0.0007	0.0009	−0.0154***
	(0.0012)	(0.0035)	(0.0052)	(0.0047)	(0.0051)	(0.0031)
ROA	0.1191**	0.0574	−1.0945***	−0.8171*	0.0800	1.8416***
	(0.0484)	(0.1321)	(0.1978)	(0.4555)	(0.1653)	(0.1544)
GROWTH	−0.0061	−0.0132	−0.0297	−0.0385*	−0.0210	0.0356
	(0.0065)	(0.0175)	(0.0225)	(0.0201)	(0.0180)	(0.0268)
BOARD	0.0124	0.1395**	−0.4348***	−0.2245	0.1621**	−0.1202**
	(0.0199)	(0.0554)	(0.0760)	(0.1888)	(0.0819)	(0.0485)
TOBINQ	−0.0024	0.0117*	0.0345***	0.0417**	0.0187**	0.0215***
	(0.0027)	(0.0069)	(0.0090)	(0.0166)	(0.0094)	(0.0078)
FIXED	0.0511*	0.0756	−0.3778***	−0.2479	0.1321	−0.7178***
	(0.0294)	(0.0879)	(0.0989)	(0.2114)	(0.1412)	(0.0641)
GS	−1.0070*	11.7230***	4.7240*	15.2262***	10.7699***	72.4816***
	(0.5773)	(1.5721)	(2.4304)	(2.6879)	(2.1140)	(1.9790)
GDP	0.0226*	0.0865**	0.1552***	0.2236***	0.1072	0.0051
	(0.0132)	(0.0376)	(0.0145)	(0.0834)	(0.0661)	(0.0085)
Constant	−10.4123***	−8.5671***	−9.0004***	−18.9298***	−10.6635***	−6.8266***
	(0.3215)	(0.3551)	(0.4152)	(4.1552)	(1.3217)	(0.2614)
Year/Firm FE	Yes	Yes	Yes	Yes	No	Yes
Observations	31053	31053	31151	31151	27434	31151
Adj-R^2^				0.7503	0.7533	0.2106
Kleibergen-Paap Wald rk F statistic	189.989	189.989				
Cragg-Donald Wald F statistic	214.981	214.981				

Note: *, **, and *** denote significant at 10%, 5%, and 1% levels, respectively. Column (6) includes only year fixed effects, since adding additional fixed effects would render the sample insufficient for estimation.

#### Heckman two-stage model.

We construct a Probit model for regression, first selecting the control variables in model (1), followed by using BR as the explanatory variable to obtain the Inverse Mills Ratio (IMR). Finally, the IMR is added to the regression in model (1) as a control variable. Columns (3) and (4) of Table 4 show that the BR coefficient is still significantly greater than 0, and the results support the hypothesis.

#### Propensity score matching.

We adopt the propensity score matching method for testing. Specifically, all the control variables are taken as covariates, and the propensity to match scores is calculated by a logit model, and then the samples are matched according to the principle of nearest-neighbor matching based on the propensity to match scores. Finally, the successfully matched samples are regressed. Column (5) of Table 4 indicates that the conclusions are valid.

#### Placebo test.

There is a possibility that the correlation revealed by the baseline regression may simply be a placebo effect. In this paper, a placebo test is used to rule out this possibility. First, all values of the BR variable are extracted, then the values are randomized into each firm-year sample, and the regression is rerun. If there is a placebo effect, then the randomly assigned BR variable is still significantly positively associated with corporate innovation. Column (6) of Table 4 indicates that the coefficient on BR is not significant. Therefore, the conclusions are robust.

#### Other robustness tests.

First, we replace the way of measuring dependent variables to conduct a robustness test. We measure the number of patents based on the actual year in which they were granted and take the natural logarithm after adding 1 to finally obtain the variable PAT2. Meanwhile, R&D investment is likewise a common indicator of firm innovation [63]. The ratio of R&D investment to total assets (RD) is used to replace the corporate innovation measure. The coefficients of BR are positive in columns (1)-(2) of Table 5, supporting the conclusions.

**Table 5:** Other robustness tests.

	(1)	(2)	(3)	(4)	(5)	(6)
VARIABLES	PAT2	RD	PAT	PAT	PAT	PAT
BR	0.0443**	0.0005**		0.0159**	0.0606***	
	(0.0182)	(0.0002)		(0.0078)	(0.0191)	
ILBR			0.5089**			
			(0.2533)			
L.BR						0.0492**
						(0.0204)
SIZE	0.4622***	−0.0012***	0.5029***	0.1716***	0.5051***	0.4835***
	(0.0271)	(0.0003)	(0.0294)	(0.0124)	(0.0280)	(0.0320)
LEV	−0.0063	−0.0002**	0.0037	0.0014	0.0022	0.0062
	(0.0045)	(0.0001)	(0.0048)	(0.0023)	(0.0047)	(0.0058)
ROA	−0.3424**	0.0054***	0.0601	0.0614	0.0986	0.1033
	(0.1484)	(0.0021)	(0.1571)	(0.0696)	(0.1577)	(0.1675)
GROWTH	−0.0284*	0.0002	0.0232	−0.0007	−0.0190	−0.0131
	(0.0160)	(0.0002)	(0.0187)	(0.0076)	(0.0170)	(0.0190)
BOARD	0.0660	0.0016*	0.1990**	0.0460	0.1340*	0.1003
	(0.0729)	(0.0009)	(0.0802)	(0.0330)	(0.0738)	(0.0847)
TOBINQ	0.0131	0.0008***	0.0110	−0.0061	0.0126	0.0086
	(0.0083)	(0.0001)	(0.0092)	(0.0038)	(0.0088)	(0.0097)
FIXED	0.2703**	0.0025*	0.1264	−0.0596	0.0480	0.0095
	(0.1307)	(0.0015)	(0.1379)	(0.0635)	(0.1249)	(0.1473)
GS	10.8909***	0.2352***	11.7979***	2.2549***	11.0180***	10.5880***
	(1.8144)	(0.0274)	(2.0523)	(0.8363)	(1.9119)	(2.1611)
GDP	0.1180*	−0.0000	0.0616	0.0010	0.0222	0.0722
	(0.0606)	(0.0006)	(0.0658)	(0.0282)	(0.0964)	(0.0685)
Constant	−10.2669***	0.0402***	−10.1243***		−9.4273***	−9.7336***
	(1.2084)	(0.0126)	(1.3051)		(1.7915)	(1.3851)
Year/Firm FE	Yes	Yes	Yes	Yes	Yes	Yes
Observations	31151	31151	26595	30198	31148	26031
Adj-R2	0.7627	0.8204	0.7534		0.7539	0.7551

Note: *, **, and *** denote significant at 10%, 5%, and 1% levels, respectively.

Second, we have replaced the measurement methods of the independent variable. We use fund data to measure the biodiversity risks at the industry level (ILBR). Referring to Chen et al. [54] and Giglio et al. [2], we constructed an annual industry-level biodiversity risk exposure indicator (BR) based on industry exposure information from 40 environmental and biodiversity funds. First, information on the positions of 40 funds directly related to biodiversity and environmental protection was collected using the AKSHARE database for the period from 2011 to 2023. Then, equations (2) and (3) are constructed. Where *wI t,m* is the weight of industry I in the market portfolio at time t, and *wI t,f* is the weight of the industry I in fund f at time t. The industry’s biodiversity risk exposure indicator is equation (3), and N is the number of funds holding industry I stocks. A large *BioRis*I t indicates that the fund’s allocation to industry I is underweighted relative to its weight in the market portfolio, i.e., industry I is more exposed to biodiversity risk. The results are shown in column (3) of Table 5, supporting the conclusions.


$$BioRis_{t,f}^{I}=w_{t,m}^{I}-w_{t,f}^{I}$$ (2)

$$BioRis_{t}^{I}=\sum_{f}^{F}BioRis_{t,f}^{I}/N$$ (3)

Third, given the large number of zero values for the number of firms’ patent applications, logarithmic transformation may increase the estimation bias, and thus, this paper attempts to utilize a panel Poisson model for the regression. The results are presented in column (4) of Table 5, supporting the conclusions.

Fourth, we add industry and city fixed effects, and the results are presented in column (5) of Table 5. The BR coefficient is positive.

Fifth, we lag the explanatory variables by one period (L.BR) and regress them again. Column (6) of Table 5 shows that the coefficient of L.BR is significantly positive.

## Potential mechanism analysis

Theoretical analysis indicates that biodiversity risk affects firms’ innovation by influencing firms’ operating costs and analysts’ attention. We construct the following model to verify the above mechanism. Where M_i,t_ are the mechanism variables, the rest are consistent with model (1).


$$M_{i,t}+\beta_{\text{0}}+\beta_{\text{1}}BR_{i,t}+\beta_{\text{2}}Controls_{i,t}+YEAR+FIRM+\varepsilon_{i,t}$$ (4)

$$PAT_{i,t}=\beta_{\text{0}}+\beta_{\text{1}}BR\times M_{i,t}+\beta_{\text{2}}BR_{i,t}+\beta_{\text{3}}M_{i,t}+\beta_{\text{4}}Controls_{i,t}+YEAR+FIRM+\varepsilon_{i,t}$$ (5)

We use the ratio of operating costs to total assets to measure firms’ operating costs (OC). Then we measure analyst attention (AA) as the value of the number of forecasters tracking listed companies plus one, and taking the natural logarithm. Column (1) of Table 6 shows that the coefficient on BR is significantly positive. This indicates that biodiversity risk raises the operating costs. The moderating effect of operating costs is further reported in column (2) of Table 6. The coefficient of BR × OC is significantly positive, indicating that biodiversity pushes firms to innovate by raising operating costs. Hypothesis 1a is confirmed.

**Table 6:** Mechanism tests.

	(1)	(2)	(3)	(4)
VARIABLES	OC	PAT	AA	PAT
BR	0.0139***	−0.0066	0.0432***	−0.0559
	(0.0047)	(0.0377)	(0.0143)	(0.0497)
BR × OC		0.1588***		
		(0.0614)		
BC		0.2091***		
		(0.0648)		
BR × AA				0.0610***
				(0.0222)
AA				0.0309*
				(0.0170)
SIZE	−0.0666***	0.5217***	0.4912***	0.4174***
	(0.0086)	(0.0271)	(0.0195)	(0.0358)
LEV	−0.0156***	0.0042	0.0169***	−0.0009
	(0.0010)	(0.0046)	(0.0035)	(0.0052)
ROA	−0.1351***	0.1576	3.4123***	0.0464
	(0.0429)	(0.1599)	(0.1363)	(0.2124)
GROWTH	0.0721***	−0.0384**	−0.0227	−0.0237
	(0.0042)	(0.0177)	(0.0145)	(0.0202)
BOARD	0.0145	0.1336*	0.1015*	0.1307
	(0.0205)	(0.0767)	(0.0577)	(0.0980)
TOBINQ	0.0115***	0.0097	0.1623***	−0.0128
	(0.0021)	(0.0089)	(0.0070)	(0.0104)
FIXED	0.1227***	0.0024	−0.1532*	0.1519
	(0.0362)	(0.1322)	(0.0886)	(0.1543)
GS	1.0431**	11.5032***	−1.1524	11.0636***
	(0.4834)	(1.8775)	(1.4444)	(2.3850)
GDP	0.0099	0.0879	0.0349	0.0902
	(0.0181)	(0.0550)	(0.0451)	(0.0840)
Constant	1.8022***	−11.0458***	−10.4074***	−8.6353***
	(0.3829)	(1.1497)	(0.8883)	(1.6766)
Year/Firm FE	Yes	Yes	Yes	Yes
Observations	29730	29730	21622	21622
Adj-R2	0.7503	0.7513	0.6182	0.7733

Note: *, **, and *** denote significant at 10%, 5%, and 1% levels, respectively.

Column (3) of Table 6 shows that biodiversity risk attracts analysts’ attention. Column (4) further reports the moderating effect of analyst attention with a significantly positive coefficient on BR × AA, indicating that analyst attention is an important channel through which biodiversity risk drives firms to innovate. Hypothesis 1b is supported.

## Heterogeneity analysis

### Heterogeneity analysis based on investor sentiment

It has been demonstrated that investor sentiment significantly affects corporate decision-making [64]. Specifically, higher investor sentiment can reduce firms’ financing constraints [65] and provide resources to support long-term decisions of firms facing external risks. Meanwhile, strong investor sentiment can drive firms to innovate to meet investor demand [66]. Based on this, this paper refers to Baker and Wurgler [67] and constructs a composite index to measure investor sentiment (IS). Then, the sample is divided into two groups of high (IS-H) and low (IS-L) sentiment according to the annual industry averages and regressed separately. Table 7 Columns (1) and (2) show that the positive correlation between biodiversity risk and firm innovation is more significant among firms with high investor sentiment. This also indicates the importance of investor decisions for corporate sustainability.

**Table 7:** Heterogeneity analysis.

	(1)	(2)	(3)	(4)	(5)	(6)
	IS-H	IS-L	HSGT = 1	HSGT = 0	HI	NHI
VARIABLES	PAT	PAT	PAT	PAT	PAT	PAT
BR	0.0682***	0.0340	0.0560**	0.0464	0.0279	0.1141***
	(0.0257)	(0.0284)	(0.0228)	(0.0350)	(0.0229)	(0.0327)
SIZE	0.5131***	0.4211***	0.4598***	0.4432***	0.5863***	0.4142***
	(0.0306)	(0.0457)	(0.0335)	(0.0614)	(0.0314)	(0.0478)
LEV	−0.0035	0.0104	−0.0050	0.0165**	0.0022	−0.0049
	(0.0058)	(0.0077)	(0.0056)	(0.0076)	(0.0054)	(0.0091)
ROA	0.1072	0.0721	0.1910	−0.1783	0.2178	0.0484
	(0.1933)	(0.2674)	(0.1989)	(0.2568)	(0.1926)	(0.2689)
GROWTH	−0.0076	−0.0151	−0.0318	0.0670*	−0.0427*	−0.0076
	(0.0273)	(0.0244)	(0.0196)	(0.0354)	(0.0224)	(0.0269)
BOARD	0.1590*	0.2057*	0.1454	0.1263	0.2603***	−0.0314
	(0.0936)	(0.1105)	(0.0930)	(0.1235)	(0.0885)	(0.1285)
TOBINQ	0.0037	0.0340	0.0144	−0.0100	0.0095	0.0038
	(0.0097)	(0.0207)	(0.0099)	(0.0210)	(0.0101)	(0.0171)
FIXED	0.1132	0.1256	0.1599	−0.2683	0.1415	0.0225
	(0.1572)	(0.1887)	(0.1621)	(0.2105)	(0.1556)	(0.2111)
GS	10.1986***	11.3923***	11.2712***	10.4911***	13.5456***	1.7187
	(2.3544)	(3.1214)	(2.4281)	(2.9298)	(2.2250)	(3.3120)
GDP	0.1180*	0.0852	0.0871	0.0950	0.0421	0.1378
	(0.0702)	(0.0878)	(0.0760)	(0.0845)	(0.0639)	(0.1350)
Constant	−11.3237***	−8.9897***	−9.6270***	−9.3029***	−11.2795***	−9.8999***
	(1.3979)	(1.8270)	(1.5412)	(2.0525)	(1.3478)	(2.6075)
Difference in coefficients between groups	0.012**		0.010***		0.000***	
Year/Firm FE	Yes	Yes	Yes	Yes	Yes	Yes
Observations	16485	13690	23498	7620	18439	12677
Adj-R2	0.7507	0.7540	0.7555	0.7041	0.7407	0.7263

Note: *, **, and *** denote significant at 10%, 5%, and 1% levels, respectively.

### Heterogeneity analysis based on capital market liberalization

Capital market liberalization has assumed an essential position in the sustainable development of emerging countries [68]. Meanwhile, capital market liberalization has implications for the relationship between biodiversity and corporate innovation as global environmental governance is increasingly emphasized. One of the most direct effects of capital market liberalization is the expansion of sources of corporate finance. However, foreign investors entering China’s domestic capital market mainly come from countries or regions with more stringent requirements on environmental information regulation, and these investors are more concerned about corporate environmental information [69]. Therefore, for firms facing natural environmental risks, there is a stronger willingness to release positive signals through innovation to cater to foreign investors [70]. We set the dummy variable HSGT based on the Shanghai-Hong Kong and Shenzhen-Hong Kong Stock Connect policies. These policies allow foreign investors to invest in Chinese stocks. We take the value of 1 for the sample in the year when the stocks are formally transferred into the stock connect and thereafter, and 0 for the opposite. The samples are regressed into subgroups accordingly. Columns (3) and (4) of Table 7 show that the driving effect of biodiversity risk on corporate innovation is more pronounced in regions with liberalized capital markets.

### Heterogeneity analysis based on industry nature

Industry heterogeneity tends to be accompanied by differences in firms’ demand and willingness to innovate. Specifically, firms in high-tech industries are more inclined to consistently and highly allocate corporate resources to corporate innovation R&D activities to maintain their business performance and market competitiveness [71]. However, non-high-tech industries have a lower need for R&D innovation because their core competencies do not rely on innovation activities [72]. Therefore, the marginal contribution of biodiversity risk to firm innovation may be relatively low. Based on this, we divide the sample into two groups of firms belonging to high-tech industries (HI) and non-high-tech industries (NHI) for the regression. Columns (5) and (6) of Table 7 show that the driving effect of biodiversity risk on firm innovation is more pronounced for non-high-tech industries.

## Distinguishing innovation strategies and types

Corporate innovation strategies can be categorized into substantive and strategic innovations [73]. Biodiversity risks are often perceived by the public as chronic and long-term risks [27], putting long-term pressure on firms. This may have a greater impact on firms’ innovation strategies. Therefore, this paper further analyzes the influence of biodiversity risk on firms’ innovation strategies. Based on this, we refer to Bi et al. [73] to measure firms’ substantive innovation (SSI) by the number of invention patent applications and strategic innovation (STI) by the total number of utility model and design patent applications. Considering that there may be a large number of zero values in the data, all variables are added by 1, and the natural logarithm is taken. The results in Table 8 show that the innovation-enhancing effect of biodiversity risk is more often reflected in substantive innovation.

**Table 8:** Distinguishing innovation strategies and types.

	(1)	(2)
VARIABLES	SSI	STI
BR	0.0400**	0.0271
	(0.0175)	(0.0190)
SIZE	0.4681***	0.4360***
	(0.0265)	(0.0260)
LEV	0.0028	−0.0018
	(0.0042)	(0.0045)
ROA	0.0046	0.1594
	(0.1401)	(0.1562)
GROWTH	−0.0244	−0.0047
	(0.0155)	(0.0164)
BOARD	0.1389**	0.1075
	(0.0692)	(0.0750)
TOBINQ	0.0208***	0.0127
	(0.0079)	(0.0085)
FIXED	0.0258	0.2283*
	(0.1167)	(0.1275)
GS	13.3949***	8.6186***
	(1.8281)	(1.9400)
GDP	0.1603***	0.0271
	(0.0562)	(0.0584)
Constant	−11.9218***	−8.4365***
	(1.1731)	(1.1656)
Year/Firm FE	Yes	Yes
Observations	31151	31008
Adj-R2	0.7354	0.7264

Note: *, **, and *** denote significant at 10%, 5%, and 1% levels, respectively.

## Conclusion

This study examines whether and how biodiversity risk affects firms’ innovation using data from Chinese A-share-listed firms from 2007 to 2022. First, we find that biodiversity risk increases firms’ innovation output. Meanwhile, we use instrumental variables, propensity score matching, and Heckman two-stage regression to support the findings. Second, this study shows that biodiversity risk pushes firms to innovate by increasing their operating costs and attracting analyst attention. Third, the positive impact of biodiversity risk on corporate innovation is more pronounced among firms with high investor sentiment, high openness of capital markets, and non-high-tech industries. This indicates that biodiversity risk drives firms to innovate, but with slight variations across scenarios. Finally, the promotion of innovation by biodiversity risk is manifested more in substantive innovation.

Based on the above findings, we put forward the following recommendations.

First, the Convention on Biological Diversity [12] indicates that a principal barrier to achieving biodiversity conservation in developing countries is the shortage of financial resources. Accordingly, governments should increase funding for industries characterized by high biodiversity risk and for ecologically fragile regions. For example, governments could establish biodiversity funds to support firms’ green technology innovations, especially focusing on non-high-tech firms. Secondly, given the evidence that financial analysts can positively influence corporate innovation and biodiversity-related disclosure, governments should strengthen capacity building for analysts. For instance, this could involve promoting awareness of biodiversity conservation policies and facilitating exchange programs for learning about biodiversity finance operations. Thirdly, governments should optimize the capital market environment. Research shows that market openness and investor sentiment can amplify the positive relationship between biodiversity risk and corporate innovation. Therefore, it is recommended that governments comprehensively incorporate biodiversity risks into financial regulations and green finance standards. On the one hand, financial institutions should be required to systematically identify, quantify, and manage biodiversity risks associated with their investment and financing activities, and these should be included in their disclosure requirements. On the other hand, differentiated regulatory incentives and policy tools can be used to guide the allocation of financial resources toward innovative projects that engage in nature conservation and sustainable use.

This study also has some limitations. First, we only focus on the impact of biodiversity risk perception on firms’ innovation, but due to data constraints, we have not yet paid attention to whether different levels of biodiversity threats faced by firms make a difference in firms’ innovation decisions, which can also be further explored in the future. Second, non-listed firms may differ from listed firms regarding firm size, access to capital, and regulatory pressure, but owing to the difficulty of obtaining data and poor data quality of non-listed firms, non-listed firms have not been taken as the object of research. In the future, case studies and field research can be used to investigate whether the threat of biodiversity risk to non-listed firms changes innovation decisions. Third, although we use an instrumental variable and multiple robustness checks, there remains a possibility that more innovative firms are systematically more likely to disclose biodiversity-related issues, which could bias the measurement of biodiversity risk exposure.

## Supporting information

S1 Appendix Description of funds related to biodiversity and environmental protection. (DOCX)
