# Supplementary material for: Biodiversity risks and corporate innovation: Evidence from China
Source: PLoS One. 2026 Mar 18;21(3):e0342348. doi: 10.1371/journal.pone.0342348 (PMC12998866; doi:10.1371/journal.pone.0342348)
Supplement: S1 Appendix — (DOCX) [file pone.0342348.s001.docx]

**Appendix**

Biodiversity lexicon is constructed based on the framework of Giglio et al. (2025), incorporating the following relevant terms: biodiversity, ecosystem(s), ecology/ecological, habitat(s), species, (rain)forest(s), deforestation, fauna, flora, marine, tropical, freshwater, wetland, wildlife, coral, aquatic, desertification, carbon sink(s), ecosphere, and biosphere.

**Table A1**

**Description of funds related to biodiversity and environmental protection**

| Code | Name | Description |
| --- | --- | --- |
| 100056 | Fortune Low Carbon Environmental Protection Mixed Securities Investment Fund | The fund mainly invests in listed companies that are engaged in or benefit from the theme of low-carbon environmental protection, and seeks to achieve returns in excess of the performance benchmark for fund shareholders through stock selection and risk control. |
| 001856 | E Fund Environmental Theme Mix A | / |
| 001166 | CCB Environmental Protection Industry Fund A | The fund is an equity fund that seeks to generate excess returns and long-term capital appreciation by selecting high quality listed companies that contribute to environmental protection, subject to effective risk control. |
| 000409 | Penghua Environmental Protection Industry Equity Fund | The fund is an equity-type fund that selects high quality listed companies in the environmental protection industry, subject to effective risk control, and seeks to generate excess returns and capital appreciation over the long term. |
| 160634 | Penghua CSI Environmental Protection Industry Index (LOF) A | The fund closely tracks its underlying index and seeks to minimize tracking deviation and error, aiming for an average daily tracking deviation of less than 0.35% and an annual tracking error of less than 4%. |
| 004925 | Changxin Low Carbon Environmental Protection Sector Quantitative Fund | The fund seeks to outperform its benchmark through active portfolio management and quantitative risk control, and by investing in high quality companies in the low carbon and environmental sectors. |
| 007163 | Axa Spdb Environmental New Energy A | The fund invests mainly in stocks of environmental protection and new energy related industries, seizing the investment opportunities of China’s green economy transformation and upgrading, taking fundamental analysis as a basis, and striving for long-term and stable appreciation of the fund’s assets under the premise of strict risk control. |
| 012504 | Guotai CSI Environmental Protection Industry 50 ETF C | The fund seeks to minimize tracking deviation and tracking error by investing primarily in the target ETF and closely tracking the underlying index. |
| 016387 | Forever Win Low Carbon Environmental Protection Intelligent Mix | The fund invests primarily in assets related to low carbon and environmental themes and seeks to achieve steady growth in net asset value over the medium to long term while controlling portfolio risk. |
| 016061 | Great Wall Environmental Protection Theme Mix C | The fund focuses on investments in listed companies with environmental themes and seeks to achieve long-term capital growth while controlling risk through a combination of quantitative and qualitative active strategies. |
| 019032 | E Fund Environmental Protection Theme Mix C |  |
| 021610 | Guotai CSI Environmental Protection Industry 50 ETF E | The fund invests primarily in the target ETF and tracks the underlying index closely, seeking to minimize tracking deviation and tracking error. |
| 000696 | China Universal Environmental Protection Sector Equity Fund | The fund pursues a bottom-up strategy based on fundamental analysis and invests primarily in high quality listed companies in the environmental protection industry and seeks to achieve sustainable and steady growth of the Fund's assets under the premise of scientific and rigorous risk management. |
| 001064 | GF CSI Environmental Protection ETF Connect A | The fund invests primarily in the target ETF and tracks the underlying index closely, seeking to minimize tracking deviation and tracking error. |
| 001975 | Invesco Great Wall Environmental Advantage Stock Fund | Based on the research results of the research team of Jingshun Great Wall, the Fund will continue to explore listed companies with environmental advantages and share their green, efficient and sustainable growth in the context of China’s economic growth to achieve long-term capital appreciation of the Fund’s assets. |
| 512580 | GF CSI Environmental Protection ETF | The fund invests primarily in the target ETF and tracks the underlying index closely, seeking to minimize tracking deviation and tracking error. |
| 002984 | GF CSI Environmental Protection ETF Connect C | The fund invests primarily in the target ETF and tracks the underlying index closely, seeking to minimize tracking deviation and tracking error. |
| 002259 | Penghua Healthy Environment Mix | The fund is a hybrid fund that seeks to achieve excess returns and long-term capital appreciation through active and flexible asset allocation and the selection of healthy and environmentally friendly thematic stocks, while effectively controlling risk. |
| 159861 | Guotai CSI Environmental Protection Industry 50 ETF | The fund invests primarily in the target ETF and tracks the underlying index closely, seeking to minimize tracking deviation and tracking error. |
| 012503 | Guotai CSI Environmental Protection Industry 50 ETF Connect A | The fund invests primarily in the target ETF and tracks the underlying index closely, seeking to minimize tracking deviation and tracking error. |
| 015060 | China Energy Saving and Environmental Protection Stock C | The fund mainly invests in energy-saving and environmentally-friendly listed companies, and seeks to achieve steady and long-term appreciation of the fund's assets under the premise of effective management of investment risks. |
| 015685 | Penghua CSI Environmental Protection Industry Index | The fund closely tracks the underlying index and seeks to minimize tracking deviation and tracking error, aiming to keep the average daily tracking deviation within 0.35% and the annual tracking error within 4%. |
| 000158 | Fullgoal Low Carbon Environmental Protection Mix (Back-end) | The fund invests primarily in quoted companies engaged in, or benefiting from, the low-carbon environmental theme and seeks to outperform its performance benchmark for the fund's shareholders through stock selection and risk management. |
| 001616 | Harvest Environmental Low Carbon Stock Fund | The fund invests mainly in stocks related to environmental protection and low-carbon industries, taking advantage of investment opportunities arising from the transformation and upgrading of China's economy, and seeks to achieve long-term and stable appreciation of the Fund's assets under the premise of strict risk control. |
| 398051 | Zhong Hai Environmental New Energy Mix | Against the backdrop of deteriorating global environmental conditions, gradual climate warming and increasing pressure on China's energy conservation and emission reduction, the fund has adopted environmental protection as its investment theme, focusing on environmental protection and new energy industries with environmental responsibility and awareness, as well as industries and enterprises with growth potential and competitive advantages in environmental protection, in order to take full advantage of investment opportunities arising from the development of the environmental protection and new energy industries in the country, and to select individual stocks, seeking long-term appreciation of the fund's assets through active weighting. Combined with active weighting allocation, the fund aims to achieve long-term stable capital appreciation. |
| 163114 | Sws Mu CSI Environmental Protection Industry | The fund uses an index-based investment approach and aims to control the average daily tracking error between the fund's NAV growth rate and its benchmark performance of no more than 0.35% and the annual tracking error of no more than 4% through strict investment process constraints and quantitative risk management tools in order to achieve effective tracking of the underlying index. |
| 164304 | New China CSI Environmental Protection Industry | The fund uses a passive indexing methodology to achieve effective tracking of the underlying index through rigorous investment procedures and quantitative risk management tools. Under normal market conditions, the fund aims to control the average daily tracking error between the net value growth rate of the fund and the performance benchmark to no more than 0.35% and the annual tracking error to no more than 4%. |

Note: The fund holding data is sourced from <https://akshare.akfamily.xyz/>. AKShare is a Python-based financial data interface library.
